# Constrictive Pericarditis: There Is Nothing More Deceptive than an Obvious Fact

**DOI:** 10.3390/diagnostics14232642

**Published:** 2024-11-23

**Authors:** Daniele Masarone, Dario Catapano, Luigi Falco, Sabrina Siniscalchi, Emilio di Lorenzo

**Affiliations:** Department of Cardiology, AORN dei Colli-Monaldi Hospital, 80131 Naples, Italy

**Keywords:** constrictive pericarditis, differential diagnosis, transient constriction

## Abstract

Introduction: we discuss the clinical case of a patient referred to our cardiology unit to evaluate the need for a pericardiectomy due to constrictive pericarditis. Imaging: the echocardiographic assessment confirmed all diagnostic criteria for constrictive pericarditis; however, we conducted a cardiac MRI before referring the patient to the cardiac surgeon. This imaging technique not only confirmed the constrictive pathophysiology but also indicated extensive pericardial inflammation, consistent with transient constriction. Clinical implications: this finding enabled us to initiate appropriate anti-inflammatory treatment, resulting in gradual clinical and instrumental improvements. Through this case, we aim to highlight the necessity of assessing the chronicity of the condition in all patients with constrictive pericarditis to determine the suitable treatment: surgical intervention for chronic cases and medical therapy for transient ones.

**Figure 1:**
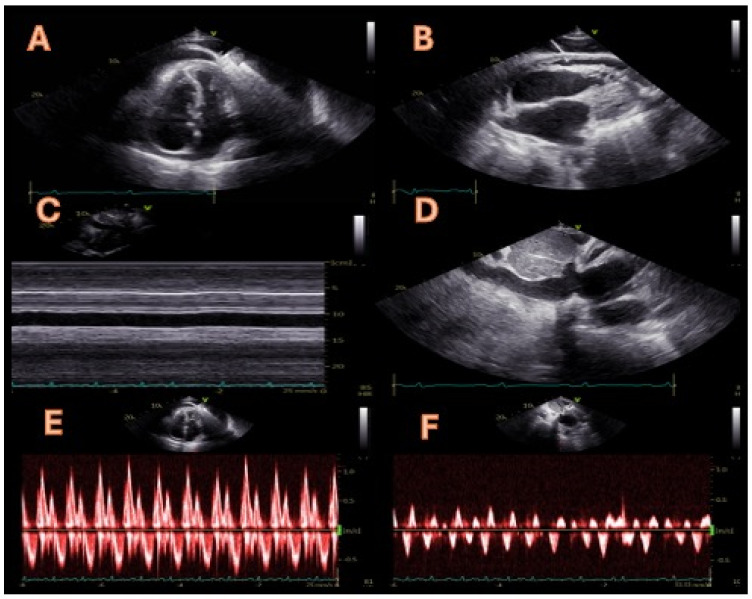
A 72-year-old man with a diagnosis of constrictive pericarditis was referred to our cardiology unit in April 2024 to assess his eligibility for a pericardiectomy. The previous diagnosis of constrictive pericarditis was based on symptoms of volume overload (peripheral oedema and weight gain) with typical features on a transthoracic echocardiogram (TTE). Upon admission, the TTE showed paradoxical motion of the interventricular septum during early diastole (septal bounce, panel (**A**)) and moderate pericardial effusion (panel (**B**)). Septal bounce is an echocardiographic sign of ventricular interdependence, serving as one of the diagnostic criteria for constrictive pericarditis identified by the Mayo Clinic [1]. Additionally, both bidimensional and M-mode assessments showed a dilated inferior vena cava that exhibited no motion during spontaneous breathing or forced inspiration (sniff), as illustrated in panels (**C**,**D**). These observations indicate inferior vena cava plethora, which may also occur in volume overload conditions or cardiac tamponade; therefore, if present, it is not included in the Mayo Clinic’s diagnostic criteria due to its low specificity and sensitivity. Lastly, a pulsed Doppler evaluation of mitral flow displayed a decline in the peak mitral E-wave velocity of over 25% during the initial inspiration alongside a prolonged isovolumic relaxation time (panel (**E**)). Meanwhile, a pulsed Doppler assessment of the hepatic veins revealed a characteristic W-shaped pattern, indicating significant diastolic flow reversals during the expiration and dilation of the hepatic veins (panel (**F**)). These last two observations are characteristic of constrictive pericarditis and confirm the diagnosis for this patient in conjunction with the abnormal septal movement.

**Figure 2:**
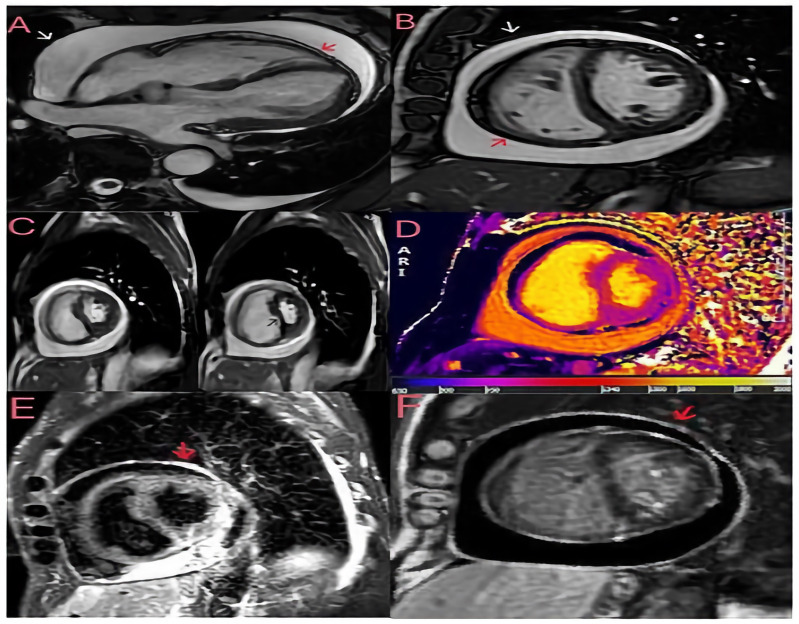
Cardiac magnetic resonance imaging (CMRI) was performed to assess the patient’s pericardial disease prior to referral to cardiac surgery. Breath-hold steady-state free precession (SSFP) cine of bright blood horizontal long axis (**A**) and midventricular short axis (**B**) views with evidence of a large pericardial effusion without compression of the ventricles in late diastole. The visceral (red arrow) and parietal (white arrow) pericardial layers are slightly thickened (3 mm). (**C**) Free-breathing SSFP cine in real time showing ventricular interdependence as a sign of constrictive pathophysiology; the left panel was taken during expiration, and the right panel during inspiration, showing septal bounce (black arrow) and crushing of the left ventricular chamber during inspiration. (**D**) The native T1 mapping sequence (modified look-locker inversion recovery) shows increased T1 times in the pericardium. (**E**) A short tau inversion recovery (STIR) T2 sequence showing pericardial hyperintensity (red arrow) compatible with oedema. (**F**) A late gadolinium enhancement (LGE) phase-sensitive inversion recovery (PSIR) sequence showing increased signal intensity in the pericardial layers (red arrow). The finding of a large pericardial effusion without tamponade and the widespread inflammation on the tissue characterisation images suggest subacute pericarditis with transient constrictive physiology, which accounts for 9–17% of cases of constrictive pericarditis and may resolve spontaneously within a few months [2,3]. Consequently, the patient was started on anti-inflammatory treatment with 600 mg of ibuprofen three times daily and 1 g of colchicine daily, resulting in clinical improvement, normalisation of inflammatory markers and discharge to home. During the follow-up, ibuprofen was progressively tapered to discontinuation, and at the last follow-up visit in October 2024, the patient was asymptomatic with no signs of volume overload and a normal plasma protein C level. This clinical case highlights the importance of assessing the chronicity of the disease by imaging in patients with constrictive pericarditis to differentiate between chronic and transient constriction, as acute/subacute cases may respond to medical therapy. Even though TTE is the imaging modality of choice for haemodynamic assessment, tissue characterisation with CMR is essential to diagnose the different phases of the pericardial inflammatory process, helping to differentiate an acute/subacute process (STIR+ and LGE+) from a chronic one (LGE+ and STIR-). Also of fundamental importance is the ability of cardiac magnetic resonance imaging to better assess the extent of pericardial effusion and pericardial thickening (a thickness > 3 mm tends to indicate chronic constrictive pericarditis). Such differentiation is crucial for tailored therapy; indeed, pericardiectomy is required in appropriate patients with chronic constrictive pericarditis [4]. In contrast, anti-inflammatory therapy is preferred in patients with transient constrictive pericarditis [5].

## Data Availability

All relevant data are within the manuscript.
